# Supplementary material for: Organic bipolar transistors
Source: Nature. 2022 Jun 22;606(7915):700–5. doi: 10.1038/s41586-022-04837-4 (PMC9217747; doi:10.1038/s41586-022-04837-4)
Supplement: Supplementary file 1 — Supplementary Figs. 1–4 and Table 1. [file 41586_2022_4837_MOESM1_ESM.pdf]

nature portfolio

https://doi.org/10.1038/s41586-022-04837-4

Supplementary information

Organic bipolar transistors

In the format provided by the authors and unedited

Supplementary Information

Organic bipolar transistors

Wang et al.

Shu-Jen Wang[1†], Michael Sawatzki[1†], Ghader Darbandy[2], Felix Talnack[3], Jörn Vahland[1], Marc Malfois[4], Alexander Kloes[2], Stefan Mannsfeld[3], Hans Kleemann[1], Karl Leo[1,3*]

[1]Dresden Integrated Center for Applied Physics and Photonic Materials (IAPP), Technische Universität Dresden, Nöthnitzer Str. 61, 01187 Dresden, Germany
[†]These authors contributed equally to this work
*email: karl.leo@tu-dresden.de

[2]NanoP, TH Mittelhessen, University of Applied Science, Wiesenstrasse 14, 35390 Gießen, Germany

[3]Center for Advancing Electronics Dresden (cfaed), Technische Universität Dresden, Helmholtz Str. 18, 01069 Dresden, Germany

[4]NCD-SWEET beamline, ALBA synchrotron light source, Barcelona, Spain

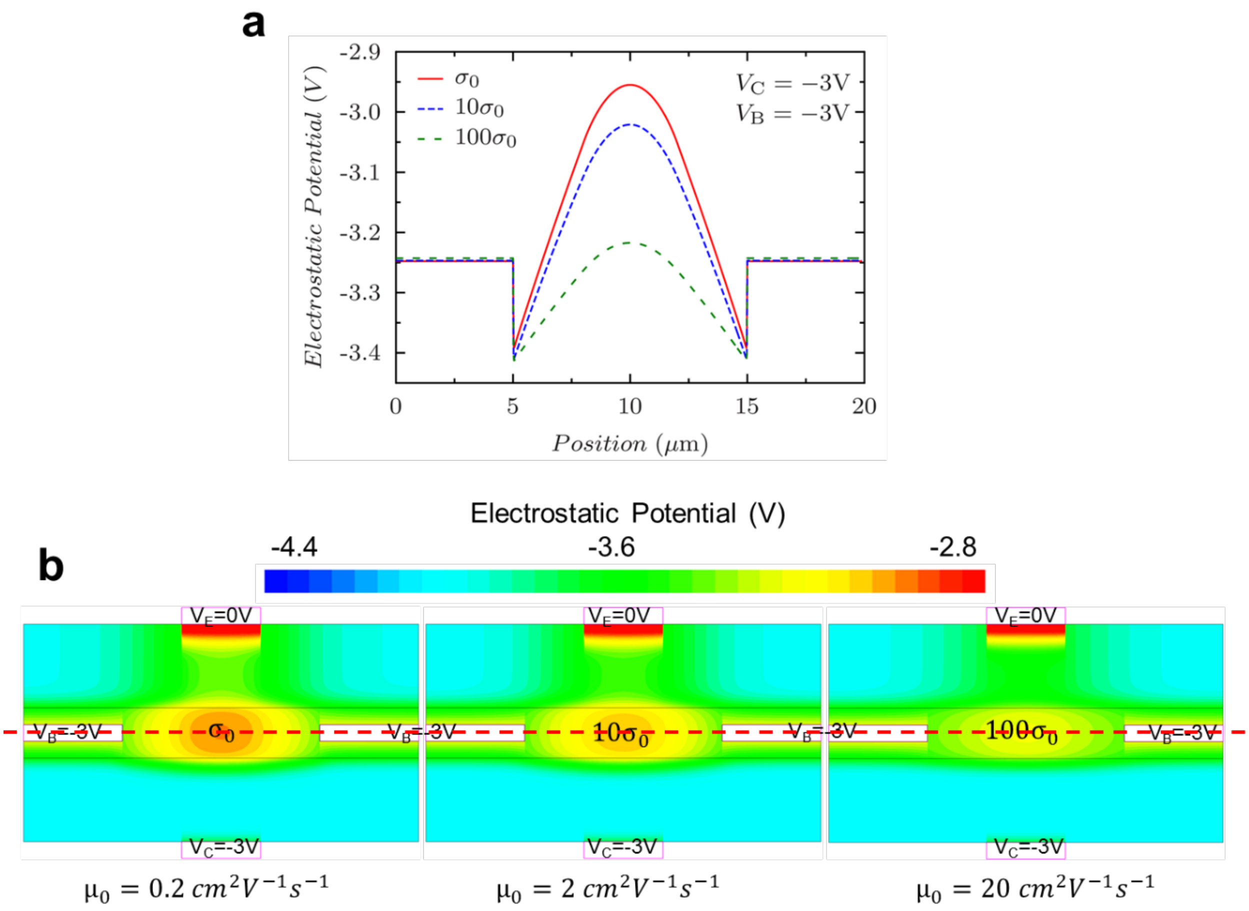

Fig. S1. (a) Electrostatic potential profile through the cutline (the dash-line indicated in red in Fig. S1b) with different base layer conductivity, $\sigma_0 = qn\mu_0$ based on the calibrated simulator to the experimental data (b) The corresponding TCAD simulation of the electrostatic potential distribution in the OBJT devices with varying base layer conductivity σ_0 through mobility μ_0.

The electrostatic potential profiles and distributions (Fig. S1) show the impact of conductivity on the potential comparing that at the base electrode meatal, with that in the organic semiconductor. Please note that we set the color map (blue is associated to -4.4V, and red for -2.8V) to see the potential distribution in the interested region (base-OSC-layer) in a more visible way. A difference between the applied V_B=-3V and the indicated potential (-3.25V) in the base electrode is caused by the fermi level of the base material The conductivity $\sigma_0 = qn\mu_0$ of the base OSC layer has been changed by considering different mobility μ_0 (see the legend) for the minority carrier "hole" in the layer. The conductivity controls and tunes the potential shape, penetration, and influence into the base-OSC-layer (see Fig. S1a). Therefore, the base control over the channel and accordingly the emitter-collector leakage current can be significantly controlled and suppressed by the conductivity. However, it is not just the conductivity, but also the applied biases, device dimensions/design and in particular the spacer between the base and emitter electrodes need to be optimized. Future work will focus on additional investigations and a more advanced optimization of the fabricated devices.

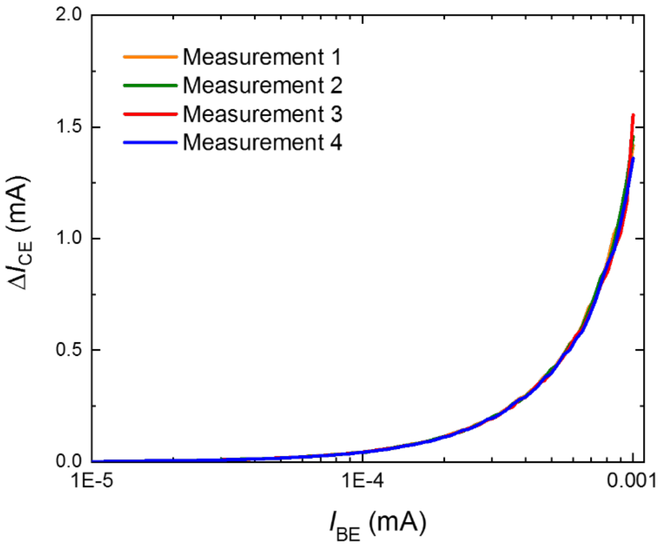

Fig. S2. Repeated OBJT amplification measurements for Fig. 1g experiments.

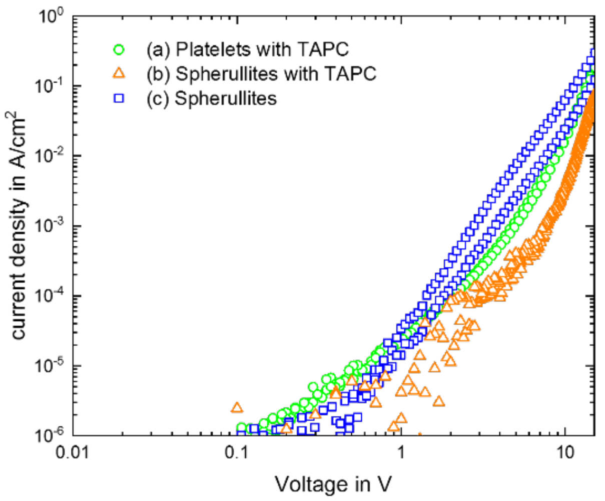

Fig. S3. *IV* characteristics of undoped rubrene films in spherulitic (with and without 5 nm TAPC) and platelet (with 5 nm TAPC) phases: Stack consists of 30 nm of undoped seed and 370 nm of undoped bulk film between Au-electrodes (active area of 100 μm×100 μm). Note that in particular the spherulite samples show a certain hysteresis.

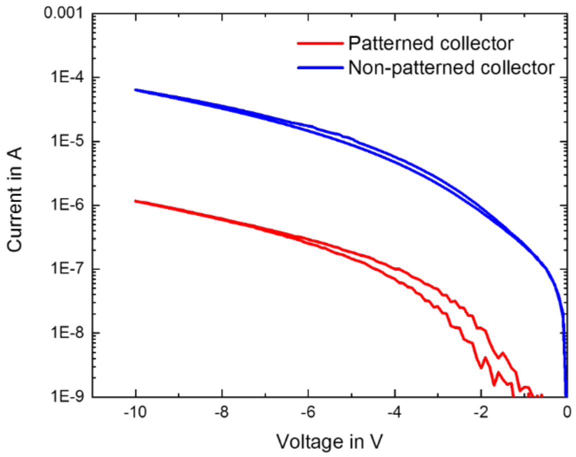

Fig. S4. Reverse current of the base-collector diodes of the OBJT devices (20 nm base thickness) with and without the structuring of the collector electrode.

Table S1. TCAD Simulation: Parameters and Units used for OBJT TCAD Simulation

**Quantity**	**Parameter Values and Units**
Device Width	1 μm
Emitter/Collector Electrode Thickness	30 nm
p_OSC Thickness	100 nm
n_OSC Thickness	30 nm
Base Electrode Thickness	10 nm
p-doping	8e17 cm^{-3}
n-doping	1e16 cm^{-3}
Low field Mobility (hole) μ_{0-hole}	2 cm^2 V^{-1} s^{-1}
Low field Mobility (electron) μ_{0-elec}	3 cm^2 V^{-1} s^{-1}
Temperature	300 K
Base Lengths L_B (Fig. 2d)	25, 10, 1, 0 μm
Distance between Adjacent Base-Electrodes L_{BB} (Fig. 2d)	50 μm
Emitter and Collector Lengths (Fig. 2d)	100, 70, 52, 50 μm
Base Length L_B (Fig. 2e)	0 μm
Distance between Adjacent Base-Electrodes L_{BB} (Fig. 2e)	100 μm
Emitter Lengths (Fig. 2e)	100, 98, 96, 90 μm
Collector Length (Fig. 2e)	100 μm
Offsets between Base and Emitter L_{BE} (Fig. 2e)	0, -1, -2, -5 μm
Base Length L_B (Fig. 2f)	25 μm
Distances between Adjacent Base-Electrodes L_{BB} (Fig. 2f)	100, 50, 25, 5 μm
Emitter and Collector Lengths (Fig. 2f)	150, 100, 75, 55 μm
